# Fatal Result of Cigarette Smoking

**Published:** 1888-02

**Authors:** 


### Fatal Result of Cigarette-Smoking.

The number of boys, in the cities especially, who begin to smoke cigarettes at the age of ten or eleven years is enormous. In most cases the habit is not indulged in excessively, and no serious harm is none, though growing boys cannot take nicotene without suffering from it to some extent. In not a few cases chronic disorders of the nervous system occur. The report comes from Philadelphia of a boy, eleven years of age, who died from tobacco-narcosis. He had been smoking half a dozen or more cigarettes daily for ten months.

There could be no more salutary law than one forbidding the sale of cigarettes to minors.

